# C-ViT: An Improved ViT Model for Multi-Label Classification of Bamboo Chopstick Defects

**DOI:** 10.3390/s26030812

**Published:** 2026-01-26

**Authors:** Waizhong Wang, Wei Peng, Liancheng Zeng, Yue Shen, Chaoyun Zhu, Yingchun Kuang

**Affiliations:** 1 College of Information and Intelligence, Hunan Agricultural University, Changsha 410128, China; 2312937036@stu.hunau.edu.cn (W.W.); shenyue@hunau.edu.cn (Y.S.); kyc@hunau.net (Y.K.); 2 School of Computer Science, Xiangtan University, Xiangtan 411105, China; 973019230@stu.hunau.edu.cn; 3 School of Computer Science and Engineering, Jishou University, Jishou 416000, China; 2022403972@stu.jsu.edu.cn

**Keywords:** multi-label classification of bamboo chopsticks defects, Vision Transformer, CNN feature extractor, Hard Examples Contrastive Loss

## Abstract

The quality of disposable bamboo chopsticks directly affects consumers’ usage experience and health safety. Therefore, quality inspection is particularly important, and multi-label classification of defects can better meet the refined demands of actual production. While ViT has made significant progress in visual tasks, it has limitations when dealing with extreme aspect ratios like bamboo chopsticks. To address this, this paper proposes an improved ViT model, C-ViT, introducing a convolutional neural network feature extraction module (CFE) to replace traditional patch embedding, making the input features more suitable for the ViT model. Moreover, existing loss functions in multi-label classification tasks focus on label prediction optimization, making hard labels difficult to learn due to their low gradient contribution. Therefore, this paper proposes a Hard Examples Contrastive Loss (HCL) function, dynamically selecting hard examples and combining label and feature correlation to construct a contrastive learning mechanism, enhancing the model’s ability to model hard examples. Experimental results show that on the self-built bamboo chopstick defect dataset (BCDD), C-ViT improves the mAP by 1.2% to 92.8% compared to the ViTS model, and can reach 94.3% after adding HCL. In addition, we further verified the effectiveness of the proposed HCL function in multi-label classification tasks on the VOC2012 public dataset.

## 1. Introduction

Disposable bamboo chopsticks, as a representative of traditional tableware, have gained widespread usage worldwide due to their natural, environmentally friendly, and renewable characteristics [1,2]. However, with the continuous expansion of production scale and the increasing market demand, quality issues of bamboo chopsticks have gradually emerged as a major bottleneck restricting industry development. Currently, defect inspection of bamboo chopsticks largely relies on manual visual examination, which suffers from low efficiency, high subjectivity, and a considerable risk of oversight. With the rapid advancement of deep learning technologies, automated defect detection methods based on computer vision have increasingly become a focal point of research [3,4,5].

In industrial quality inspection, automated defect identification technologies typically encompass three categories of tasks: defect classification, defect object detection, and defect segmentation. This study focuses on defect classification, particularly multi-label defect classification. With the advancement of deep learning, convolutional neural network (CNN) based methods have achieved remarkable breakthroughs in complex visual tasks. For instance, ResNet demonstrated outstanding feature extraction capability on ImageNet [6]; the YOLO series models, benefiting from a complete ecosystem, attained excellent performance and favorable configurability in defect detection scenarios [7,8,9]. In parallel, several recent works have explored large-scale appearance inspection and fine-grained visual understanding in industrial and agricultural domains. Fan et al. [10] introduced a large-scale grain appearance dataset and benchmark, highlighting the importance of fine-grained recognition and distribution-aware modeling in real-world inspection tasks. Meanwhile, the rise of Vision Transformer (ViT) [11] has driven the further application of self-attention-based models in industrial vision scenarios.

Although ViT have achieved promising results in defect detection, they still face significant challenges when processing images with extreme aspect ratios. Taking bamboo chopstick images as an example, whose aspect ratios often exceed 10:1, naively resizing them to the default ViT input size of 224 × 224 leads to severe distortion of local details, making it difficult to capture subtle defects such as black spots, mold stains, and cracks. The conventional patch embedding mechanism of ViT also struggles to provide effective positional encoding for such data.

Recent related studies have attempted to fuse CNN with ViT; however, most approaches merely incorporate CNN as front-end feature extractors in a structural manner, lacking structure optimization tailored to specific industrial scenarios. For special data such as bamboo chopstick images, which exhibit high aspect ratios and fine-grained defects, these generic fusion strategies fail to provide sufficient geometric representation capacity, thereby limiting the model’s detection performance.

There are also notable differences between multi-class and multi-label classification in data annotation. Multi-class classification addresses problems where a sample must be assigned to exactly one of several mutually exclusive classes, implying that each sample receives only a single label [12,13]. In multi-class tasks, the SoftMax activation is typically used to produce a probability distribution over classes, ensuring that each sample is associated with a single label. In contrast, multi-label classification permits a sample to be associated with multiple non-exclusive labels, making it more suitable for describing samples with multiple attributes. In multi-label tasks, the output layer usually adopts the Sigmoid activation, enabling independent probability outputs for each label and allowing a sample to possess multiple labels simultaneously [14,15].

Consequently, in the bamboo chopstick multi-label defect classification task, severe class imbalance is common; high-frequency “easy” examples tend to dominate gradient updates, making it difficult for the model to focus on more challenging hard examples. Among mainstream approaches, binary cross-entropy (BCE) is the most widely used loss; however, it suffers from the problem of easy examples dominating the gradients [16].

To address these issues, inspired by the integration of CNN and ViT, this paper proposes a Convolutional Feature Embedding (CFE) module tailored to the structural characteristics of bamboo chopstick images, and incorporates it into the ViT framework to construct an improved visual Transformer—C-ViT. Unlike previous approaches that simply combine CNN and ViT, we carefully design the parameters of the CFE module specifically for the bamboo chopstick dataset, rather than naively replacing positional encoding with conventional convolutions. Leveraging the local receptive field and translation invariance inherent in CNN, the module encodes features of images with extreme aspect ratios, thereby avoiding the geometric distortion caused by traditional patch embedding especially preserving the fine texture information of subtle defects [17,18].

Meanwhile, to mitigate training bias induced by the dominance of “easy” examples in multi-label classification, this paper proposes a Hard Examples Contrastive Learning (HCL) loss function. HCL dynamically identifies hard examples whose prediction confidence is close to the decision threshold, and constructs a contrastive learning objective based on their label and feature similarity. This mechanism forces the model to enhance its discriminative capability for the subtle defect features present in hard examples.

To address this issue, this paper proposes a Hard examples Contrastive Learning (HCL) loss function. The HCL dynamically identifies hard examples whose prediction confidence is close to the decision threshold, and constructs a contrastive learning objective based on their label and feature similarities. This mechanism compels the model to enhance its discriminative capability for subtle defect features in hard examples.

To validate the effectiveness of the proposed method, a bamboo chopstick defect dataset (BCDD) containing 4000 samples was constructed, covering five defect types: mildew, bending, black spots, cracks, and slenderness. Experimental results demonstrate that the proposed C-ViT model combined with the HCL achieves a mean Average Precision (mAP) of 93.8% on the test set, significantly outperforming existing benchmark models. Furthermore, the generalization capability of the HCL was also verified on public datasets.

The main contributions of this paper are summarized as follows:

(1) This paper proposes an improved visual transformer model, C-ViT, which introduces the CFE module for bamboo chopstick defect classification, which can better handle image samples with extreme aspect ratios (such as bamboo chopsticks).
(2) A new loss function, Hard Examples Contrastive Loss (HCL), is proposed to enhance the model’s ability to dynamically selects hard examples. Extensive experiments are conducted on self-built and public datasets. For example, on the public dataset VOC2012, the mAP can reach 92.8%.
(3) We construct a bamboo chopstick defect multi-label classification dataset (BCDD), which contains 4000 defective samples, with no less than 500 samples in each defect category.

## 2. Related Work

### 2.1. Defect Detection

Research on industrial defect detection has optimized deep learning models for metals, glass bottles, and other domains. For example, Wei et al. [19] combined the receptive field attention convolution and contextual broadcast median to propose an improved RFAConv-CBM-ViT model to address the high variability and sample imbalance of metal defects. Cheng et al. [20] implemented a cascaded hybrid approach combining YOLOv5 and ViT for high-precision detection and classification of pipeline defects. Experiments on a laboratory-simulated dataset demonstrated that the proposed method improved classification accuracy by 15% compared to single-model approaches while maintaining detection efficiency. This hybrid strategy provides a novel and efficient solution for pipeline safety inspection. Tomita et al. [21] developed a machine learning method specifically for detecting the threaded regions on glass bottle surfaces. By focusing on the threaded area rather than the entire bottle surface, this approach overcomes the accuracy loss caused by reflections in traditional inspection methods.

### 2.2. CNN–ViT Hybrid Vision Models

CNN excel at feature extraction, while ViT model global dependencies; their integration has become a research focus. The Convolutional Vision Transformer (CvT) [22] introduces a hierarchical Transformer with convolutional token embeddings and convolutional Transformer blocks using convolutional projections. This architecture leverages the strengths of both CNN and ViT and further demonstrates that positional encodings can be safely removed in existing vision models. ConViT [23] incorporates a gated positional self-attention mechanism, allowing a “soft” convolutional inductive bias within the self-attention framework, resulting in a ViT architecture that exhibits convolution-like properties [24].

### 2.3. Multi-Label Classification and Loss Function Design

Multi-label classification allows each sample to be associated with multiple non-exclusive labels, and its key challenges include class imbalance and the difficulty of modeling label correlations. ALS [25] introduced a novel loss function, the Asymmetric Loss (ASL), which effectively alleviates the imbalance between positive and negative samples in multi-label classification by treating them differently. This loss function dynamically down-weights easy negative samples and filters out potentially mislabeled ones. Moreover, ASL can be extended to single-label classification and object detection tasks. The algorithm is easy to implement and does not increase training complexity. Focal Loss [26] mitigates the severe foreground–background class imbalance by modulating the standard cross-entropy loss, effectively down-weighting well-classified samples. This mechanism forces the model to concentrate on the sparse set of hard examples, preventing the learning process from being dominated by abundant and easily classified negative samples. Suchetti et al. [27]. proposed a multi-label classification approach that combines fuzzy logic with deep neural networks to address the class imbalance commonly encountered in multi-label learning. They further introduced symbolic representation of time series data to reduce dimensionality, thereby improving model performance and flexibility in time-series classification tasks. Liu et al. [28] proposed a concise and efficient multi-label classification method based on Transformer decoder. By using the label embedding as the query vector, the cross-attention mechanism of the decoder is used to adaptively extract the local features related to different categories, and the accurate recognition of multiple objects in the image is realized. The method only needs the standard Transformer architecture and visual backbone network, and its concise structure and excellent performance make it a new benchmark for multi-label classification tasks. Gao et al. [29]. developed an efficient mineral recognition model based on multi-label image classification. They constructed a mineral feature dataset by simulating real-world conditions and innovatively employed the Query2Label framework combined with a MaxViT-T feature extraction network and the Asymmetric Loss function. Unlike approaches based on loss re-weighting and label-relation modeling, MSL [30] introduces mask-driven contextual learning at the feature level, enhancing recognition of small and occluded objects.

## 3. Materials and Methods

### 3.1. Datasets

#### 3.1.1. Bamboo Chopsticks Defects Dataset

**Figure 1:**
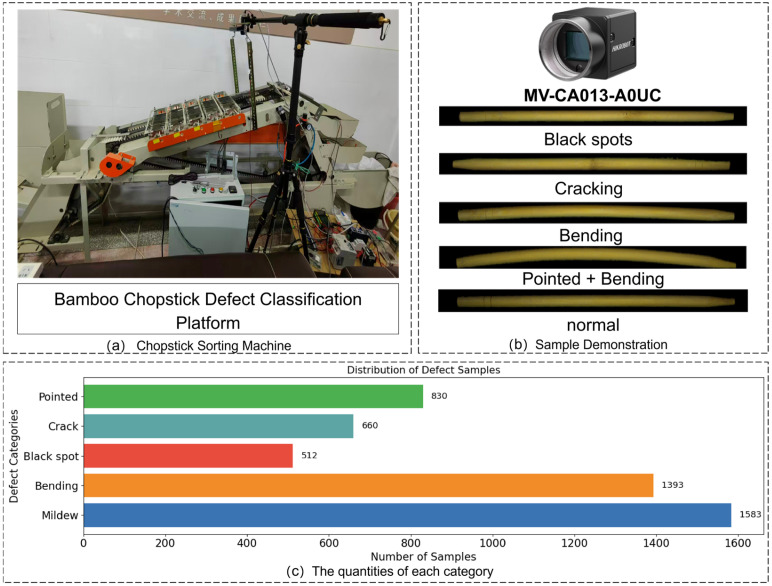
Data distribution map.

Data collection is the foundation of building an effective machine learning model. The BCDD was collected from an actual bamboo chopstick production line, as shown in Figure 1a. The dataset is designed for multi-label classification of disposable bamboo chopstick defects and includes five defect categories: mildew, bending, black spots, cracking, and pointed. Each chopstick may contain one or more defect types simultaneously. Both the data collection and validation processes are based on this machine platform.

The dataset contains a total of 4000 defective samples, with no fewer than 500 samples per defect category, as illustrated in Figure 1c. Each sample has a resolution of 1500 × 60 pixels. Given the high aspect ratio of bamboo chopsticks, we employed an anisotropic resizing strategy (512 × 96) to maximize pixel occupancy of valid regions, thereby preserving structural integrity in the 128 × 24 feature representations and enhancing the capture of fine-grained defects while minimizing computational overhead. Figure 1b presents representative samples of different bamboo chopstick defects, where the second-to-last row illustrates typical cases of composite defects, and the last row shows normal bamboo chopstick samples. The dataset is divided into training, validation, and test sets in a ratio of 7:2:1. To enhance generalization, data augmentation techniques such as horizontal flipping, vertical flipping, and translation were applied to increase sample diversity.

In this study, an industrial camera (MV-CA013-A0UC) was fixed above the conveyor belt using a support bracket. During operation, the conveyor gear triggered image capture at six different angles for each chopstick. A complete mathematical synchronization model was established to coordinate the chopstick’s rotation and the camera’s sampling frequency, ensuring that each chopstick’s potential defects were fully captured. All images were standardized to 60 × 1500 mm in physical dimensions for consistent learning. Data labeling strictly followed the national standard GB19790.2-2005, ensuring annotation accuracy and consistency. Through these meticulous steps, a comprehensive bamboo chopstick defect dataset containing mildew, bending, black spots, cracking, and pointed defects was constructed, providing a solid foundation for automatic defect recognition and classification.

#### 3.1.2. VOC2012 Dataset

**Figure 2:**
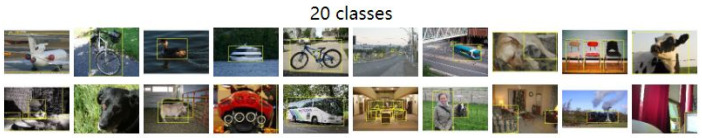
VOC2012 Classification Chart.

The VOC2012 dataset is a standard visual dataset widely used in image understanding tasks, mainly for the research and evaluation of object detection and semantic segmentation tasks. The dataset contains a total of 11,540 RGB images, covering a variety of daily life scenes. The images come from the Internet and real-life shooting, and are highly general and representative. VOC2012 provides high-quality pixel-level semantic segmentation annotations for 20 object categories, including common ground scene targets such as “people”, “animals (such as dogs, horses, sheep, etc.)”, “vehicles (such as cars, airplanes, trains, etc.)”, “furniture and plants”, etc., as shown in Figure 2. The VOC2012 dataset covers samples with different lighting, postures, occlusions and background complexities, which provides strong support for the generalization ability and robustness evaluation of the model. In this study, the VOC2012 dataset is used to verify the generalization performance of the proposed model.

### 3.2. Method

#### 3.2.1. The ViT Network

ViT is a model proposed by the Google team in 2020 to apply Transformer to image classification. However, because its model is “simple” and has good effects and strong scalability, it has become a milestone in the application of transformers in the field of CV. The ViT model consists of three modules: Embedding layer, Transformer Encoder, and MLP Head.

The Embedding layer splits the input image into a series of fixed-size image blocks. After each block is flattened, it is mapped to a D-dimensional vector through a linear projection layer, and a learnable position encoding is superimposed to enable the model to perceive the position relationship of the image blocks. Finally, Class Token is added as a global feature fusion to generate an N+1-dimensional sequence containing position information. Transformer Encoder consists of L stacked Transformer blocks, each of which is mainly composed of Layer Norm, Multi-Head Attention, Dropout, and MLP Block. Feature extraction is achieved through a serialized processing flow. First, the input image sequence is layer-normalized, and then input into the efficient multi-head self-attention layer. The long-range dependency of the image is calculated through the query, key, and value matrices, and Dropout is used to enhance the generalization of the model. The attention output is added to the original input through the residual connection method, and the layer is normalized again. Then, the high-level features are extracted through the MLP layer, and the results are connected to the input residual again and normalized. The MLP Head extracts the Class Token vector finally output by the Transformer Encoder and maps it to the category probability through the multi-layer perceptron MLP.

#### 3.2.2. The C-ViT Network

Considering the extreme aspect ratio (1500 × 60) of the bamboo chopstick dataset and the difficulty of hard examples learning by the model, we propose an improved ViT model, which is tailored for the bamboo chopstick recognition scenario.

**Figure 3:**
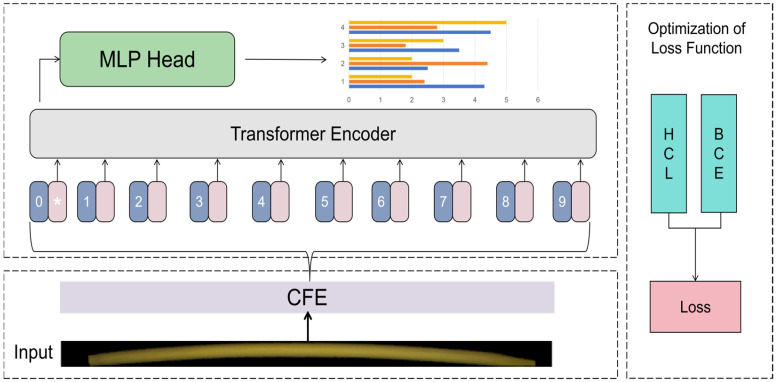
Methodology Overview Chart.

As shown in Figure 3, there are two main improvements in this paper, firstly, the CNN Feature Extractor (CFE) module is introduced to replace the traditional patch embedding module in ViT, and the input image is converted into a feature representation suitable for ViT model processing, so as to better adapt to the particularity of the data and improve the recognition accuracy of the model. Secondly, in view of the current multi-label classification loss function mainly focusing on the probability prediction optimization of the label itself, but not paying attention to the problem that the labels that are difficult to distinguish are difficult to learn effectively by the model due to the low contribution of the gradient, this paper proposes Hard Examples Contras Loss (HCL) to construct a comparative learning mechanism through the correlation between dynamic difficult case selection and labels and features, so as to enhance the model’s ability to discriminate hard examples and enhance the robustness of the model. This architecture not only inherits the advantages of ViT in efficient image modeling, but also significantly improves the model’s ability to process special structures in bamboo chopstick images. The design principles and core details of the proposed CFE will be elaborated in Section 3.2.3, and the relevant formulas for HCL will be elaborated in Section 3.2.4.

#### 3.2.3. CNN Feature Extractor Module

**Figure 4:**
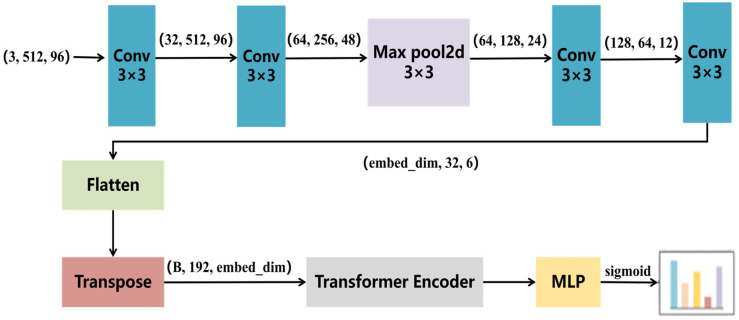
Structure of the C-ViT model.

The Vision Transformer (ViT) model relies on dividing the input image into several patches and generating an embedding vector through linear projection. However, in the bamboo chopstick defect classification task studied in this paper, the input image has a large aspect ratio (512 × 96). Directly using the traditional Patch Embedding method may have the following problems: (1) The patch size does not match the image size: For images with relatively small height and large width, fixed-size patch division may lead to insufficient feature extraction or information loss. If the image size is processed to 224 × 224, the image will be severely distorted, thus affecting the final prediction result; (2) Insufficient local feature capture capability: Traditional Patch Embedding lacks the ability of convolutional layers to extract local spatial features and is not sensitive enough to subtle defects in detection tasks. In order to solve the above problems, this study proposes a method of replacing Patch Embedding with CNN. This method can not only efficiently extract the local features of bamboo chopstick defects through the local perception capability of CNN, but also adapt to the input requirements of Transformer Encoder through the downsampling process of multi-layer convolution. The specific design is as follows:

The C-ViT model structure combined with CFE is shown in Figure 4. We propose to use the CNN Feature Extractor (CFE) module to replace the traditional Patch Embedding. Specifically: First, use two 3 × 3 convolutional layers to perform preliminary feature extraction on the input image, and then use the pooling operation (MaxPool2D) to downsample the feature map. Finally, the feature map is projected to the specified embedding dimension through two 3 × 3 convolutional layer to form a two-dimensional feature sequence that ViT can process.

Through the aforementioned design, the initial features with dimensions (3, 512, 96) undergo convolution and pooling operations, ultimately resulting in a feature map of size (embed_dim, 32, 6), where embed_dim depends on the chosen base model. This feature map is then converted into the sequence format (B, 192, embed_dim) required by the Transformer Encoder via Flatten and Transpose operations, where B represents the batch size. Compared to the traditional ViT, the C-ViT offers the following advantages: (1) Adaptability to input data of varying sizes; (2) CNN has stronger local feature extraction ability, which is more suitable for extracting small target features of bamboo chopsticks surface defects.

#### 3.2.4. HCL Function

In the training process of multi-label classification tasks, due to the imbalance of the number of categories and the unclear features of individual categories, it may be difficult to extract the features of some categories, resulting in easy-to-classify samples dominating the training process. To solve this problem, this section proposes an HCL function suitable for multi-label classification tasks. The method consists of two core parts: (1) a hard examples selection mechanism based on prediction error; (2) constructing positive and negative sample pairs based on the similarity of hard examples labels, and designing a contrast loss function based on feature similarity. In addition, we jointly train HCL with the classic Binary Cross-Entropy (BCE) loss, which effectively improves the recognition ability of hard examples while maintaining model stability.

In multi-label classification, due to the large number of labels and the large differences between them, the difficulty of predicting different labels is often uneven. Some labels are easily predicted by the model due to their obvious visual features, while other labels become blind spots for model learning due to the small number of samples and unclear features. Therefore, we designed a hard examples selection mechanism based on the error between the predicted and true labels to dynamically select the categories that are difficult for the model to predict correctly during training.

Suppose the predicted probability matrix output by the model is $P\in R^{B\times C}$, The true label is $Y\in \{0\;,1{\}}^{\;B\times C}$, Where B is the batch size and C is the number of labels. We calculate the prediction error as:

(1) D=|P−Y|

For each label category, select the first k=⌊B·topk_ratio⌋ samples with the largest prediction error as the “hard examples” of the current category. Generate a mask matrix $H\in \{0,1{\}}^{B\times C}$ to specify the location of the hard examples for subsequent contrast loss calculations.

The advantage of this hard examples selection mechanism is that it allows the model to pay more attention to those label dimensions that have not been “learned well” during training, avoiding the training process being dominated by a large number of easy-to-classify samples.

Through the hard examples selection mechanism, we obtain hard examples. However, simply locating hard examples is not enough, because hard examples often represent that the sample is rare or the features are not obvious, so simply increasing the weight may not learn useful feature representations. Therefore, in the process of constructing the loss function, we introduced label similarity and feature similarity to assist the learning of hard examples through categories with similar semantics or features.

In multi-label classification tasks, a sample may have multiple labels at the same time, and there may be semantic associations between different labels. The label of each sample can be represented as a binary set (1 if the label exists, 0 otherwise). Jaccard similarity measures the degree of overlap of two sets by calculating the intersection and union ratio of the two sets, which is naturally suitable for this binary representation. Therefore, we use Jaccard similarity to measure the label similarity between samples:

(2) $$S_{label}[i,j]=\frac{\left|y_{h}^{\left(i\right)}\cap y_{h}^{\left(j\right)}\right|}{|y_{h}^{\left(i\right)}\cup y_{h}^{\left(j\right)}|+\epsilon }$$

Among them, $y_{h}$ represents the label subset extracted from the hard examples. We set a similarity threshold $. If the label similarity between two samples is greater than this value, they are judged as a positive sample pair $P_{i}=\{j|S_{label}[i,j]\geq \delta \}$, otherwise they are a negative sample pair $N_{i}=\{j|S_{label}[i,j]<\delta \}$.

In order to measure the distance between samples in the feature space, we use the feature vectors extracted by the backbone network to construct a feature similarity matrix. First, we normalize the extracted features and then perform dot product calculations:

(3) $${\tilde{f}}_{i}=\frac{f_{i}}{{\left\|f_{i}\right\|}_{2}}$$

(4) $$S_{feat}[i,j]={\tilde{f}}_{i}\cdot {\tilde{f}}_{j}^{T}$$

Among them, $f\in R^{M\times D}$ is the feature vector, M is the number of hard examples, and D is the feature dimension.

Based on the positive and negative sample pairs and feature similarities obtained above, we use InfoNCE contrast loss to supervise the samples, which can not only aggregate semantically related hard examples, but also increase the distance between dissimilar categories in the feature space, thereby effectively alleviating confusion between categories:

(5) $$L_{HCL}=\frac{1}{M}\sum_{i=1}^{M}-log\frac{\sum_{j\in P_{i}}\exp(S_{feat}[i,j]/\tau )}{\sum_{j\in P_{i}\cup N_{i}}\exp(S_{feat}[i,j]/\tau +\epsilon }$$

Among them, τ is the temperature coefficient, and ϵ is a small constant to prevent division by zero. Finally, we combine the above contrast loss with the traditional binary cross entropy loss function to construct the final loss function:

(6) $$L_{total}=L_{BCE}+\lambda \cdot L_{HCL}$$

Among them, $L_{BCE}$ is the traditional BCE loss and λ is a hyperparameter that controls the contrast loss weight.

The HCL function is introduced not only to pick out hard examples so that model training is not dominated by easily classified samples, but also to allow the model to learn the representation of these hard examples more reasonably in the semantic and feature space, thereby further improving their recognition accuracy.

#### 3.2.5. Chapter Summary

This chapter proposes two core improvement methods for the problems of the ViT model in the multi-label classification task of bamboo chopstick defects. First, in the feature extraction part, the traditional ViT model has the problems of patch size mismatch and insufficient local feature extraction when processing extreme aspect ratio images. Therefore, the C-ViT model is proposed, which adapts the Transformer Encoder input through the CFE module and effectively captures the local features of subtle defects. Secondly, in order to solve the problem of unclear features and class imbalance of hard examples in multi-label classification, the HCL contrast loss is proposed. This loss function combines the hard examples selection mechanism with the label and feature dual similarity to construct the contrast loss, and optimizes the model with BCE. This method effectively enhances the model’s prediction ability for hard examples and alleviates the problem of easy-to-classify samples dominating the training process.

## 4. Experimental Results and Analysis

### 4.1. Experimental Environment and Parameter Settings

The operating system used in the experiment is Windows 10, and the AMD Ryzen 9 7950X processor is paired with the NVIDIA GeForce RTX 4090 graphics card, with 128G memory. The deep learning framework uses Pytorch 2.0.1 version, and the CUDA version is 12.0. In terms of model training, the experimental settings are as follows: the input image resolution is adjusted to 512 × 96 pixels, the batch size is set to 64, the optimizer uses Adam and sets the initial learning rate to 1 × 10^−4^, the similarity threshold is δ 0.5, the contrast loss weight λ is 0.5, and the total training cycle is 300 epochs. The specific experimental running environment is detailed in Table 1.

### 4.2. Evaluation Indicators

In order to effectively evaluate the improvement effect of the model, this experiment uses three indicators: mean average precision (mAP) of all categories, overall F1 score (OF1), and average F1 score of each category (CF1). The specific calculation formula is as follows:

(7) $$Precision=\frac{TP}{TP+FP}$$

(8) $$Recall=\frac{TP}{TP+FN}$$

(9) $$mAP=\frac{1}{N}\sum_{i=1}^{n}AP_{i}$$

(10) $$OF1=\frac{2\cdot Precision\cdot Recall}{Precision+Recall}$$

(11) $$CF1=\frac{1}{N}\sum_{c=1}^{n}\frac{2\cdot Precision_{c}\cdot Recall_{c}}{Precision_{c}+Recall_{c}}$$

Among them, TP represents the number of samples with defects that are correctly detected; FP represents the number of samples without defects but misjudged as defects; FN represents the number of samples with defects but not detected; N represents the total number of defect categories in the detection task; Precision and Recall represent the overall detection accuracy and recall rate, respectively; AP represents the integrated area under the Precision and Recall curves, which is used to comprehensively evaluate the detection performance.

### 4.3. Experimental Results on BCDD

To verify the effectiveness of the improved C-ViT and other methods on the BCDD, this section designed comparative experiments with mainstream multi-label classification algorithms such as ViT-Small, ResNet-50, ASL (ResNet-50), and Q2L under the same equipment and training parameters, using mAP, OF1, and CF1 as evaluation metrics.

The experimental results are shown in Table 2 below. ViT-Small performed poorly on all three metrics: mAP at 91.6%, OF1 at 85.8%, and CF1 at only 81.7%, indicating that its ability to identify more difficult category labels still has room for improvement. The unmodified C-ViT achieved a mAP of 92.8%, slightly better than ResNet-50′s 92.0%. OF1 and CF1, at 87.2% and 82.8%, respectively, were comparable to ResNet-50′s 86.9% and 82.5%, indicating that the visual Transformer structure improves semantic modeling. ASL achieved a mAP of 93.6%, slightly lower than Q2L, but its performance in OF1 (89.5%) and CF1 (87.7%) was superior, demonstrating that its adaptive loss mechanism effectively alleviates the label imbalance problem. Q2L achieved a 94.1% mAP, while OF1 and CF1 reached 90.1% and 88.0%, respectively, indicating significant advantages in overall detection capability and multi-label learning strategies.

Simultaneously, we conducted comparative experiments with recent hybrid models. ConViT integrates inductive biases into the Vision Transformer through the ingenious design of gated positional self-attention units, achieving a mAP of 92.4%, with OF1 and CF1 scores of 87.0% and 82.4%, respectively. FasterViT achieves a mAP of 92.6% by introducing a hierarchical attention mechanism combined with efficient local feature extraction, while its OF1 and CF1 reach 87.1% and 82.6%, respectively.

**Figure 5:**
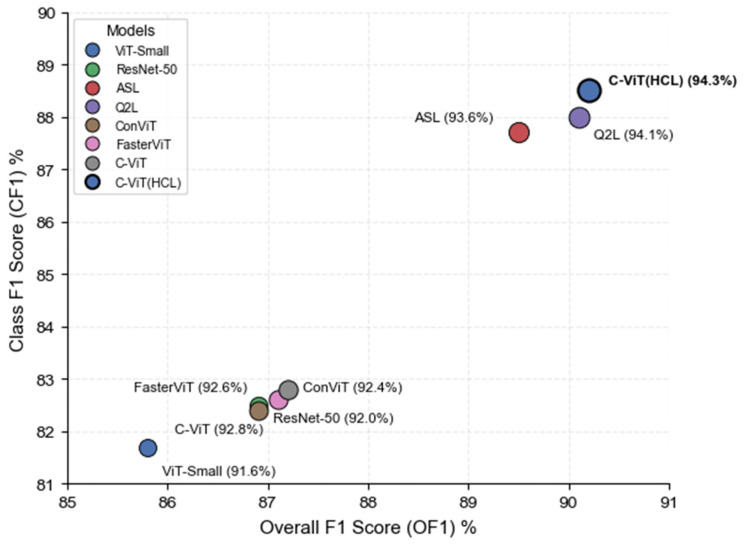
Performance comparison of different models on the BCDD in terms of OF1 and CF1 metrics.

However, as shown in Figure 5, when using HCL, the performance of C-ViT (HCL) is further improved to mAP 94.3%, which is 2.7% higher than ViT-Small’s 91.6% and 2.3% higher than ResNet-50. OF1 and CF1 are also improved to 90.2% and 88.5%, respectively, verifying the effectiveness of this method in enhancing the multi-label classification ability of the model.

### 4.4. Ablation Experiment

In order to evaluate the impact of the proposed CFE module and HCL function on model performance, this section conducts an ablation experiment based on the ViT-Small model.

First, the effectiveness of the CFE module is evaluated by evaluating whether to use the CFE model and the impact of the input image size on the model. The specific experimental results are shown in the following Table 3. We evaluated the impact of image size on the ViT model with two different image size inputs. When the input image size is 224 × 224, the mAP is only 91.6, while when the patch size in the patch embedding is modified to adapt to the bamboo chopstick input image, the mAP is 90.9, which is a decrease. Then we introduced the CFE module, and when the image size was kept at 512 × 96, the mAP was 92.8%, an increase of 1.2%. After introducing the HCL function, the mAP was 94.3%, from which we can clearly see the effectiveness of the proposed method.

In the CFE module, we employ a small number of convolutional layers for lightweight feature extraction to avoid structural redundancy and control computational overhead. The feature embedding dimension is kept consistent with the baseline model, ViT-Small, to ensure fair comparisons. To analyze the impact of key hyperparameters on performance, we conduct ablation experiments with different kernel sizes (3 × 3, 5 × 5, and 7 × 7) and pooling strategies (AvgPool and MaxPool), as summarized in Table 4.

The experimental results indicate that, under the same pooling strategy, model performance slightly degrades as the kernel size increases, suggesting that an excessively large receptive field is unfavorable for modeling fine-grained bamboo chopstick defect features. In addition, MaxPool consistently outperforms AvgPool across all configurations, demonstrating its advantage in preserving locally salient features. Considering both performance and model simplicity, the configuration with a 3 × 3 kernel and MaxPool achieves the highest mAP (92.8%) and is therefore adopted as the default setting in this study.

Then, in order to evaluate the effectiveness of the HCL function, we designed the following ablation experiments using the hard examples selection mechanism, label similarity, and feature similarity in the HCL. Among them, our models all use C-ViT. The specific experimental results are shown in the following Table 5. When the hard examples selection mechanism is used alone and then the hard examples are weighted, the mAP is improved by 0.8%. When label similarity is added, there is also an improvement of 0.2%. When we use the complete HCL, the mAP reaches 94.3%, an increase of 1.5%, which proves the effectiveness of each module in the HCL. Note that the reason why feature similarity is not designed to be used alone is that in our loss calculation, feature similarity depends on the positive and negative sample pairs obtained based on label similarity.

The effectiveness of the HCL function primarily depends on the selection of hard examples and the construction of contrastive objectives based on label and feature similarity. To further explain and validate the proposed mechanism, we conducted hyperparameter ablation experiments on the number of hard examples and the similarity threshold.

As shown in Table 6, when the Topk_ratio was set to 10%, the performance improvement of the model was not significant, indicating that selecting too few hard examples limited the formation of sufficient contrastive relationships within a batch. As the Topk_ratio increased, the model performance gradually improved, showing a slight enhancement at 20% and reaching its peak at 40%. However, when the Topk_ratio continued to increase to 60%, the performance declined. This degradation is likely due to the inclusion of excessive samples, which diluted the definition of “hard examples” and introduced easier or irrelevant samples, thereby weakening the model’s ability to focus on truly difficult instances.

As shown in Table 7. For the label similarity threshold, it plays a crucial role in constructing the contrastive loss by determining the sets of positive and negative hard examples associated with each current hard examples. As shown in Table 6, when the similarity threshold is set to 0.5, the performance improvement is marginal, likely because the threshold is too low to provide sufficient discriminability between positive and negative samples. As the threshold increases to 0.6 and 0.7, a noticeable performance gain is observed, indicating that the model benefits from a clearer separation between positive and negative pairs. However, when the threshold is further increased to 0.8, the performance drops. This degradation may result from the overly strict matching conditions induced by a high threshold, which exclude potential positive pairs and consequently undermine the adequacy of contrastive learning and the model’s generalization capability.

### 4.5. Experimental Results on Public Datasets

Since our CFE module is designed for the special size of bamboo chopstick images, our comparative experiments on public datasets only compare the HCL function. The dataset used is PASCAL VOC 2012.

As shown in Table 8, the proposed HCL function achieves the best performance on the VOC dataset. Specifically, compared with the traditional BCE loss function, the HCL function is better than the BCE loss function in these major indicators. Compared with the Foca-Loss loss function, it is improved by 1.2% on mAP, 2.19% on OF1, and 2.77 on CF1. Compared with the ASL function, it improved by 0.6% on mAP, 1.84% on OF1, and 2.47 on CF1. Experimental results show that the proposed HCL prime function has performance advantages compared with the sun loss function commonly used in multi-label classification tasks. These results suggest that HCL more effectively focuses on ambiguous or boundary-region samples, which are typically underrepresented or insufficiently weighted by existing loss functions. By integrating hard-example selection and contrastive learning guided by feature and label similarity, HCL strengthens the model’s ability to distinguish subtle differences among confusing defect patterns. Consequently, the proposed loss function demonstrates clear advantages over conventional losses widely used in multi-label classification tasks.

**Figure 6:**
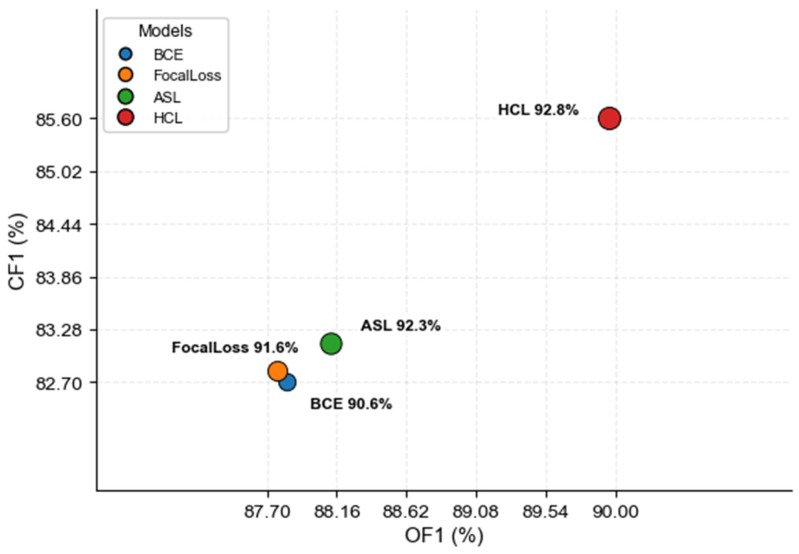
Performance comparison of different loss functions on the VOC dataset based on OF1 and CF1 metrics.

As shown in Figure 6, the visualization provides an intuitive comparison of the performance of different loss functions on the VOC dataset, clearly demonstrating that HCL achieves the best results across all key metrics.

## 5. Discussion

### 5.1. Addressing Labor Costs and Work Efficiency Issues in Traditional Methods

As the production scale of disposable bamboo chopsticks continues to expand, the traditional quality inspection process has gradually become a major bottleneck restricting production capacity and quality stability. Although manual visual inspection has long been the primary method, its limited efficiency, poor consistency, and high labor dependency have become increasingly prominent. For example, a bamboo chopstick production line can produce millions of chopsticks per day. If it relies entirely on manual inspection, even at an efficiency of 2000 chopsticks per person per hour, it would require 20 people working continuously for 25 h to complete the daily inspection task. This not only results in high labor costs and management pressure, but also causes inspectors to lose focus due to long, repetitive tasks, which increases the rate of missed detection of subtle defects, leading to quality fluctuations and potential risks.

The introduction of the C-ViT model has significantly optimized the bamboo chopstick defect detection process. By integrating this model into the production line’s automated optical inspection system, it enables real-time and efficient inspection of bamboo chopsticks, significantly reducing the need for manual inspection. The system can process hundreds of bamboo chopsticks per second, significantly exceeding manual inspection speeds without interrupting the production line. This effectively alleviates the workload of manual quality inspection, freeing personnel to focus on more critical tasks such as data analysis and quality control, thereby improving overall intelligence.

From the perspective of reducing labor costs, the deployment of this model effectively replaces traditional, high-intensity manual inspection methods. Manual inspection requires a long time to identify multiple complex defects on bamboo chopsticks under adverse conditions such as noise and strong light. This not only limits efficiency but is also susceptible to subjective factors such as emotion and fatigue. In contrast, the automated inspection system based on the C-ViT model can be linked with high-speed industrial cameras to capture high-definition images and complete defect classification in milliseconds, achieving inspection efficiency far exceeding that of traditional manual teams. Enterprises can significantly reduce the number of quality inspectors, avoid human errors, and achieve round-the-clock, standardized automated quality inspection.

### 5.2. Novel Contributions to Model Enhancement

Regarding model enhancement, for the improvement of the ViT model, previous ViT and its variants use patch embedding to positionally encode the input image to obtain the image’s position information, but most of them use images with a moderate aspect ratio, which is different from the bamboo chopstick data studied in this paper, which has an extreme aspect ratio. If the bamboo chopstick data is forcibly resized to square image data, it will cause image distortion and loss of small defect features. Inspired by the characteristics of convolution, a series of convolutions are used to extract and transform the input image, and finally obtain the input required by the ViT encoding layer. In the future, it can be considered to replace the patch embedding with traditional convolution, which can be further optimized into dynamic convolution or deformable convolution to adapt to the various complex defects and scale changes in bamboo chopsticks.

Regarding the loss function, the traditional loss function based on binary cross entropy (BCE) is very common in many defect detection studies, but it treats all samples and labels equally, resulting in high-frequency and easy-to-distinguish samples dominating the gradient update, while the contribution of hard examples (such as small targets and occluded samples) is easily ignored. To address this issue, this paper proposes the HCL function. This function dynamically selects hard examples with prediction confidence levels close to a threshold and constructs a contrastive learning objective based on hard examples labels and feature similarity. This enhances the model’s ability to extract features from defects in hard examples. Furthermore, it incorporates a dynamic focusing mechanism to enhance gradient distribution, thereby improving performance in challenging scenarios. Our experimental results demonstrate that the HCL function outperforms traditional binary cross entropy (BCE)-based loss functions. Currently, the contrastive learning component of HCL is primarily based on confidence screening and feature similarity, but in multi-label or multi-scale scenarios, inter-category relationships can be more complex. Future work could consider constructing multi-level, multi-granular positive and negative comparison pairs, combined with label semantic topological relationships or graph attention networks, to ensure that the model maintains feature discriminability in the face of multi-category interference.

## 6. Conclusions

In this paper, we propose an improved ViT model architecture; on the basis of ViT, we integrate the CFE module proposed in this paper, which is specially designed for bamboo chopstick samples with special shapes, and uses the local perception ability of convolutional neural network to efficiently extract subtle defect features on the surface of bamboo chopsticks, while avoiding the influence of image deformation on feature representation. At the same time, the HCL function is also proposed, which avoids the need for explicit modeling of complex label dependencies, and enhances the characteristic learning ability of the model for the defects of hard examples through dynamic screening of hard examples prediction of hard examples defects.

To verify the validity of the proposed method, we conducted extensive ablation studies and comparative experiments on a newly established private dataset, BCDD, and a public benchmark dataset VOC2012. The results show that each module contributes positively to classification performance, and their combination achieves significant improvements. Our final model achieved 94.3% mAP on BCDD, outperforming the original ViT and other classical baselines. In addition, experiments on VOC2012 datasets further validate the generalization ability of the proposed method.

All in all, our work provides a robust and scalable identification and classification solution for the quality inspection of bamboo chopsticks. In future work, we plan to explore and introduce lightweight deployment technology to achieve real-time detection and classification of bamboo chopsticks in field applications.

## Figures and Tables

**Table 1:** Experimental running software configuration and hardware configuration.

Parameter	Configuration
GPU	AMD Ryzen 9 7950X
CPU	NVIDIA GeForce RTX 4090
Memory	128 G
Operating System	Windows 10
CUDA	Cuda 12.0
Pytorch	Pytorch 2.0.1

**Table 2:** Comparative experiments on the BBCD dataset.

Models	mAP (%)	OF1 (%)	CF1 (%)
ViT-Small	91.6	85.8	81.7
ResNet-50 [6]	92.0	86.9	82.5
ASL [22]	93.6	89.5	87.7
Q2L [25]	94.1	90.1	88.0
ConViT [23]	92.4	86.9	82.4
FasterViT [24]	92.6	87.1	82.6
C-ViT	92.8	87.2	82.8
C-ViT (HCL)	94.3	90.2	88.5

**Table 3:** Ablation experiment.

Model	CFE	HCL	mAP (%)
ViT (224 × 224)	×	×	91.6
ViT (512 × 96)	×	×	90.9
C-ViT (512 × 96)	√	×	92.8
C-ViT + HCL	√	√	94.3

**Table 4:** CFE Module Hyperparameter Ablation Experiment.

ID	Kernel Size	Pooling	mAP (%)
A	3 × 3	AvgPool	91.9
B	5 × 5	AvgPool	91.8
C	7 × 7	AvgPool	91.6
**D**	**3 × 3**	**MaxPool**	**92.8**
E	5 × 5	MaxPool	92.4
F	7 × 7	MaxPool	92.2

Bold text is used to highlight the best method.

**Table 5:** Ablation experiment.

Models	Hard Example	Label Similarity	Feature Similarity	BCE	mAP (%)
A1	×	×	×	√	92.8
A2	√	×	×	√	93.6
A3	√	√	×	√	93.8
A4	√	√	√	√	94.3

**Table 6:** Effect of different Top-k ratios on the performance of the HCL function.

Topk_Ratio	10%	20%	40%	60%
mAP (%)	93.0	93.4	94.3	93.2

**Table 7:** Effect of different label similarity thresholds on the performance of the HCL function.

Label Similarity Thresholds	0.5	0.6	0.7	0.8
mAP (%)	92.9	93.2	94.3	93.3

**Table 8:** HCL’s comparative experiment on the VOC dataset.

Models	mAP (%)	OF1 (%)	CF1 (%)
ViT-S + BCE [27]	90.6	87.83	82.71
ViT-S + FocalLoss [23]	91.6	87.77	82.83
ViT-S + ASL [22]	92.3	88.12	83.13
ViT-S + HCL	92.8	89.96	85.60

## Data Availability

The data used to support the findings of this study are available from the corresponding author upon request.

## Abbreviations

The following abbreviations are used in this manuscript:

ViT	Vision Transformer
C-ViT	CNN Vision Transformer
CFE	CNN Feature Extractor
BCE	Binary CrossEntropy Loss
HCL	Hard Examples Contrastive Loss
ASL	Asymmetric loss
Q2L	Query2label
